# The presence of wolves leads to spatial differentiation in deer browsing pressure on forest regeneration

**DOI:** 10.1038/s41598-023-44502-y

**Published:** 2023-10-11

**Authors:** Adam Wójcicki, Zbigniew Borowski

**Affiliations:** 1 Department of Mountain Forests, Forest Research Institute, Ul. Fredry 39, 30-605 Kraków, Poland; 2 Department of Forest Ecology, Forest Research Institute, Sękocin Stary, Poland

**Keywords:** Ecology, Zoology, Ecology, Environmental sciences

## Abstract

With the recent return of large carnivores to forest ecosystems, an important issue for forest owners and managers is how large predators influence the behaviour of their natural prey and, consequently, cervid browsing pressure on forest regeneration. To investigate this issue, we analysed deer pressure on Scots pine and European beech plantations in northern Poland's ecosystems with and without permanent wolf populations. Two characteristics were used to describe deer browsing patterns in plantations: distance from the forest edge (spatial pattern of browsing) and number of saplings browsed (browsing intensity). Beech saplings were more intensively browsed by deer compared to pine saplings. In a forest ecosystem not inhabited by wolves, spatial variation in browsing patterns on small-sized beech plantations was the same between the edge and the center. In contrast, browsing pressure by deer was greater at the edges on large-sized pine plantations. The presence of wolves reduced deer browsing on beech and increased browsing on pine saplings. In addition, deer foraging behaviour changed in large-sized pine plantations, and browsing pressure increased only in the central areas of the plantations. We assume that the presence of wolves in a forest landscape is an important factor that alters browsing pressure on the youngest stands and their spatial pattern, and that this may be a major factor in stand regeneration, especially in small forest patches.

## Introduction

In recent decades, the numbers and population densities of many deer species have increased in temperate forests, including in Europe^1^. The reasons for this phenomenon lie in changes favorable to these herbivores during the twentieth century, including the disappearance of populations of large predators^1,2^. As a result, the increasing number and density of ungulates increases browsing pressure on vegetation, leading to higher losses in agricultural and forestry production^1,3^. Although it is worth noting that deer damage is not solely related to deer population density^4^. Trees and shrubs make up a significant share of the diet of wild ungulates^5,6^. Browsing of woody plants by deer is one of the major problems of modern forestry and consumes a large portion of the funds spent on tree protection in commercial forests. This problem mainly affects tree species preferred by deer, such as oaks *Quercus spp.*, hornbeams *Carpinus betulus*, silver firs *Abies alba* and Scots pines *Pinus sylvestris*^7,8^.

Intense herbivore pressure on woody and shrub vegetation, while often favoring the development of herbaceous vegetation and creating favorable conditions for the maintenance of a wider range of forest plant community species^9–11^, conflicts with timber production and hinders the achievement of forest management goals. Current ungulate densities in temperate and boreal forests have long-term effects on forest structure, composition and litter depth, implying that these herbivores can slow down natural succession and reduce the sapling richness^12^. Deer damage young trees primarily by browsing shoots and stripping bark. Damaged trees may die^13^, develop more slowly^14^, have lower biomass growth^15^, and are more susceptible to damage from invertebrate infestations^16^. Loss of their main shoots also causes trees to take on a shrub form^17^. Patches of varying sizes in forest stands are attractive foraging sites for ungulates, as they find food more easily when they emerge from the closed forest^18^. Forest edges are particularly attractive to deer and thus these animals can largely shape the vegetation within them^19^. For this reason, young forest plantations are particularly vulnerable to severe damage from these herbivores. Furthermore, abundant early successional forest stands and edge habitats, together with local high deer densities, may cause significant external threats to stands near old and mature forest communities because widely wandering deer also penetrate deeply into them^20^.

In the Northern Hemisphere, large carnivores are returning to their native ecosystems^21^. In Poland, for example, the grey wolf *Canis lupus* L. populations are increasing^22,23^. With the return of wolves to forests, the question arises whether this could reduce deer pressure on forest regeneration. Predators may indirectly affect lower trophic levels by influencing prey behaviour and reducing density^24^. They limit herbivore pressure on vegetation, which creates better conditions for plant development (a trophic cascade effect^25,26^). In forest ecosystems, large carnivores can affect forest stands in this indirect way because there is a strong relationship between the extent of tree damage caused by herbivores and stand regeneration^27,28^.

Large carnivores' predation risk can affect ungulate density, distribution and behaviour, probably operate at different spatial scales^29^, but so far the influence of wolf presence on deer feeding behaviour at different scales (landscape, stand) has been demonstrated only in the protected Białowieża primaeval forest^29–31^. It has been argued that in natural ecosystems, like in Białowieża, large carnivores may alter the foraging behaviour of browsers at fine spatial scales (stand level), which could have long-term consequences for woody plant communities and affect forest ecosystem structure and composition^32^. In contrast, recent data from commercial forests suggest that the presence of wolves in the ecosystem does not affect or even increase damage to forest plantations caused by large herbivores such as moose *Alces alces* L. and the authors of these studies suggested that the attractiveness of the food base was the stronger factor than the risk of predation for this herbivore species^33–35^. There is limited data on the spatial pattern of foraging by deer at fine scales within gaps of different sizes. In addition, Kuijper et al.^36^ concluded that in human-dominated landscapes, fear is more likely to be caused by anthropogenic disturbance (even nonlethal effects of hunter presence) than by the presence of large predators, so the intensity and spatial pattern of browsing by wild ungulates may differ from those in natural, protected ecosystems.

From a scientific and practical perspective, this knowledge is important for forest managers where deer (such as red deer *Cervus elaphus* L. and roe deer *Capreolus capreolus* L.) cause the greatest damage to young, productive stands.

The purpose of our study was to investigate whether the presence of wolves in forest ecosystems affects the intensity and spatial distribution of browsing damage by deer in pine and beech plantations, which may lead to changes in timber production and forest quality. We tested two hypotheses: (1) deer browsing intensity is lower in ecosystems where wolves are present, and (2) deer browsing intensity is higher near forest edges.

## Materials and methods

### Study area

The study was conducted between 2015 and 2017 in three forest districts (FD) in northern Poland: Borne Sulinowo Forest District (BS), Polanów Forest District (POL) and Manowo Forest District (MAN). BS is located near the town of Borne Sulinowo (53°34′52″ N 16°32′00″ E) and covers 204.32 km^2^ of flat terrain with some slightly hilly areas. The predominant tree species is pine (about 90% of the stands). POL is located near the town of Polanów (54°07′10″ N 16°41′18″ E), covering 168.32 km2 and consists of both flat and hilly terrain. Pine and European beech (*Fagus sylvatica*) are the main tree species (together about 90% of the stands). MAN is located near the town of Manowo (54°07′30″ N 16°18′06″ E) and covers 172.03 km^2^ of hilly or flat terrain. The main tree species is pine (about 84% of the stands, and 87% together with beech).

In each FD, hunting is conducted annually during the hunting season. The hunting season for red deer lasts from August 21 to February 28 (bulls), from October 1 to January 1 (does) and from January 1 to February 28 (calves), and for roe deer from May 11 to September 30 (bucks) and from October 1 to January 15 (does and fawns). In this region of Poland, 2.2–2.4 ind./km^2^/year of red deer and 2.7–2.9 ind./km^2^/year of roe deer were harvested. Also, the forests of each of these FDs are available for recreation (e.g. cycling, hiking). However, there are no tourist spots in any of them that can attract a particularly large numbers of tourists.

The detailed characteristics of the study area are shown in Table 1. Data on ungulate densities and hunting consist of unpublished 2016 data from official hunting and forestry statistics. In addition, single individuals of European bison *Bison bonasus* and moose occasionally appeared within the BS and single individuals of fallow deer *Dama dama* occasionally appeared within the MAN and POL. MAN and BS wolf populations were stable (about 1.2 individuals/100 km^2^ each) and average pack size varied between 3.5 and 5.6 individuals^37^. One wolf pack was present in MAN and two packs were present within BS. At the beginning of the study, no permanently functioning wolf packs were recorded in POL—only single wolves were observed sporadically at the end of the study period (these were usually individuals moving through the area).

**Table 1:** The characteristics of study plots.

Forest district	Wolf population	Wild ungulate density (ind./km^2^)		Study plots
		Red deer	Roe deer	
Borne Sulinowo BS	Present	6.8	7.9	12 pine plantations
Manowo MAN	Present	7.7	8.4	10 beech plantations
Polanów POL	Absent	7.5	8.8	6 pine plantations 6 beech plantations

### Field measurements

Three- to five-year-old Scots pine plantations of 1–5 ha with the same soil characteristics and forest type and three- to five-year-old round or elliptical beech plantations of about 0.1 ha were randomly selected for the study, because they were the most common forest crops within the three sites. The selected plantations met the following criteria:

1. The saplings were unfenced and unprotected against browsing;
2. Each study plot was located at least 300 m from buildings, public roads and tourist spots and was completely or mostly surrounded by older stands. There was one unpaved forest road near each study plot (closer than 50 m), which might have limited browsing pressure on saplings by making deer avoiding them^34,38,39^ and increased the wolf predation risk, as these carnivores take advantage of forest road infrastructure^40^;
3. Sapling heights ranged from 20 to 150 cm, the size most susceptible to browsing^8,13,41^. Plantations with higher saplings (> 150 cm), were not selected for the measurements because we wanted to avoid the situation when saplings become too high for deer to be browsed during the 3-year study period and the plantations become unattractive for large herbivores before the study ends. Also, up to this tree height, forest plantations are still an open environment where deer can see and perceive danger. Taller trees create an entirely different forest development medium—a forest thicket with lots of cover but little visibility.

Thirty-four study plots in total were selected for measurements (Table 1).

In addition, representatives of the genera *Rubus spp*. and/or *Vaccinum spp*. were present in each plantation, increasing the likelihood of tree browsing by deer^42,43^. There were also representatives of other vegetation groups, the most numerous of which were (in varying proportions): birch *Betula pendula* seedlings, *Calluna vulgaris*, *Juncus spp.*, *Festuca spp.*, *Poa spp*. No large grasslands were present within the POL and MAN forest districts. Within the BS were two large nature reserves (moors), but none of the study plots was closer than 500 m from these open areas.

The beech plantations had an area of 0.1 ha, while the pine plantations had an area of 1–5 ha. Because of their size, the beech plantations were divided into zones less than 10 m or more than 10 m from the nearest older forest edge. Data from the pine plantations were collected in zones that were less than 10 m from forest edge and no closer than 50 m from the nearest forest edge (and close to the plantation centre). Each year four 10 m transects perpendicular to the edge of the plantation were randomly selected in each zone. We noted the number of saplings along each transect and whether traces of fresh browsing were present on the main shoots of each sapling (the freshly browsed apical shoots were noticeably softer and lighter in colour than those previously browsed). The plantations were single-species, so we analysed only pine saplings within pine plantations and beech saplings within beech plantations. If seedlings of other tree species (e.g. birch) were found in transects, which occurred very rarely, they were not included in the analysis. Traces of browsing on side shoots were not included in our analyses, as the loss of these shoots does not cause significant changes in the saplings’ form and their growth rate. We then calculated the proportion of trees with traces of browsing on apical shoots among all measured trees per transect. In each forest plantation, fresh browsing pressure was evaluated annually from 2015 to 2017 in late spring (April–May), after the greatest winter and spring pressure from deer^44^. During the study period, neither tracks nor faeces of bison, moose or fallow deer were found within the study plots.

### Statistical analysis

To analyse the data, we used a generalized linear mixed model fitted by maximum likelihood with binomial distribution for the response variable and logit linkage function. We calculated proportion of damaged trees as the number of browsed (apical shoot damaged) and unbrowsed trees per transect in each zone of the study plot^35^. This was our indirect indicator of browsing pressure and forest regeneration success^41^. The proportion of damaged trees was used as a response variable. All predictors were also binary coded: the apical shoot (1-browsed, 0-not browsed), presence of wolves (1-present, 0-not present), distance to forest edge (edge < 10 m-1, far > 50 m (for pine)/ > 10 m (for beech)-0), and tree species (pine-1, beech-0). The study year (2015/2016/2017) and plot (specific forest plantation) were used as random factors. Initially, all interactions between dependent variables were considered: wolf presence, distance to the forest edge, and tree species, but only significant interactions were included in the final model. All statistical analyses were performed using the lmerTest package^45^ in R software^46^.

The null model contained only tree species as a predictor variable. We added more predictor variables to the null model and compared subsequent models using the likelihood ratio test to see which one fits the data better. We assessed how the model fits the data using the Hosmer–Lemeshow (HL) goodness of fit (GOF) test.

### Ethical approval

The authors declare that the research was carried out in compliance with the IUCN Policy Statement on Research Involving Species at Risk of Extinction and the Convention on the Trade in Endangered Species of Wild Fauna and Flora.

The authors declare that the research was carried out in compliance with the IUCN Policy Statement on Research Involving Species at Risk of Extinction and the Convention on the Trade in Endangered Species of Wild Fauna and Flora.

## Results

A total of 2 087 pine saplings (within 144 transects) and 1 664 beech saplings (within 128 transects) were analyzed. The mean number of saplings per transect were: 4.99 ± 1.25 (SD) within the close zone (< 10 m to the forest edge) and 8.75 ± 1.37 (SD) within the far zone (> 50 m to the forest edge) for pine, and 5.13 ± 0.77 (SD) within the close zone (< 10 m to the forest edge) and 5.80 ± 0.99 (SD) within the far zone (> 10 m to the forest edge) for beech.

Although the final model performed significantly better than the null (species only) model (Chi-squared = 13.97, *p* = 007) it did not fit data well (Hosmer–Lemeshow Goodness-of-Fit Test, Chi-squared = 30.72, df = 8, *p* = 0.0002). The reason why the model did not work well was due to two different effects. First, the presence of wolves mainly affected foraging distance (the location of foraging and thus its intensity depending on the size of the plantation, which varied between species). Second, the browsing intensity depended mainly on the tree species, hence the lack of interaction with the wolf. The interaction effect of wolf and species was insignificant in any of the interaction effects of factors.

**Figure 1:**
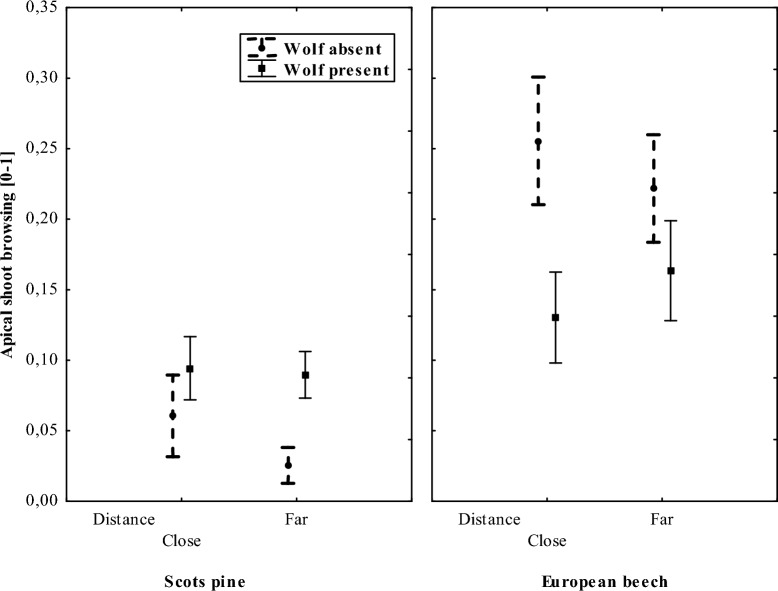
The proportion (mean ± SD) of freshly browsed apical shoots of Scotch pine and European beech saplings in different distances from the forest edge: in forests with (BS and MAN) and without (POL) permanent wolf populations.

The intensity of browsing differed between landscapes with and without wolf populations and by distance from the forest edge. In forests inhabited by wolves, browsing levels were higher than in forests without permanent wolf populations, but only for pine. For beech an opposite browsing pattern was observed (Table 2, Fig. 1). In both pine and beech plantations, deer preferred to browse near to the forest edge in landscapes with no wolves (Table 2, Fig. 1). Deer browsed pine saplings less intensively than beech saplings (Table 2). The presence of wolves increased the browsing pressure of deer on pine far from the forest edge, while a decrease in browsing close to the forest edge was observed in beech plantations.

**Table 2:** Model output results for browsing on pine and beech saplings together (with interactions).

Effects	Estimate	SE	z value	Pr( >|z|)
Intercept	− 2.0015	0.6904	− 2.899	0.00374
Wolf (Yes)	**0.7769**	**0.2883**	**2.695**	**0.00705**
Distance (Close)	**0.3521**	**0.1733**	**2.032**	**0.04211**
Species (Pine)	**− 1.7952**	**0.2788**	**− 6.440**	** < 0.001**
Wolf (Yes)*Distance (Close)	**− 0.7070**	**0.2280**	**− 3.101**	**0.00193**
Distance (Close) *Species (Pine)	0.4151	0.2249	1.845	0.06497

Wolf (Yes)—wolf present in the landscape (Yes vs. No), Distance (Close)- distance from the forest edge—above 10 m in beech and 50 m in pine plantations (Far vs. Close), Species (Pine)—analysed tree species’ saplings (pine vs. beech).

*SE* standard error, *Pr* *p* value.

Significant values are in [bold].

## Discussion

The results of a study conducted in North America show that large carnivores can have strong effects on prey populations by reducing their density and changing their behaviour, which can lead to a chain of changes at different trophic levels, i.e., cascade effects^47,48^. In our study, we hypothesised that the presence of wolves in the forest ecosystem would reduce the browsing intensity of deer in forest plantations regardless of their main sapling species. However, the results obtained partially contradicted this prediction. It was also noted that the presence of predators altered the spatial pattern of deer foraging. In pine plantations, deer tended to consume more pine saplings in the central zone. Within beech plantations, we observed a decrease in browsing at the forest edge. These results contrast with observations in Yellowstone National Park, where 15 years after the return of wolves, a decline in the deer population was accompanied by a significant decrease in the number of young trees damaged and an increase in young tree survival^49^ (but see Kauffman et al.^50^). Our results also differ from those of a study from Scandinavia, where the presence of wolves had no effect on damage caused by moose in pine plantations^35^, but they are consistent with the results of other studies in which moose browsing on pines was higher in wolf territories^33,34^. However, as has been highlighted in North America, predation on large prey is sometimes wishful thinking, while a trophic cascade may be weaker than claimed and strongly dependent on adequate sampling^51^.

So, the obvious question arises: why does the presence of wolves increase the browsing pressure of deer on saplings? According to the Optimal Foraging Theory, the most intuitive answer is that deer browsing pressure in wolf-inhabited landscapes may be concentrated in places with high food availability and good visibility, such as young and large forest plantations^52^. Such behaviour helps animals minimise foraging time and easily detect danger (predators). Some confirmation of the above explanation comes from the results of studies conducted in North America showing that deer minimise the risk of being preyed upon by coursing predators by relying on early detection, which is facilitated by the use of large-sized, open forest plantations^53,54^. Although dense vegetation cover near the forest edge may provide safety to deer by reducing detection^42^, it may obstruct visibility and escape routes, increasing predation risk from apex predators^29,31^. This would explain why distance from the forest edge was not statistically significant factor in the case of the beech plantations—these gaps were too small (0.1 ha) to provide sufficient distance for early detection of predators by deer, so they likely felt equally safe (or unsafe) within the entire plantations. We confirmed our second hypothesis that deer foraging behaviour varies spatially, but only under specific spatial conditions. However, sometimes the forest edge is the safest location^42^ or simply the most attractive location where a trade-off can be made to provide both cover for safety and open space for grazing at safer times^55^. Wolf risk is an important factor creating a landscape of fear and influencing deer foraging behaviour, even in commercial forests, where human activities (e.g., recreation, hunting) influence deer browsing behaviour at the stand and landscape level^33,36,38^ and fear is triggered by anthropogenic disturbance rather than the presence of large predators^36,56^. Although the hunting pressure within our study areas was relatively high compared to other regions of Poland, it was due to alike deer densities similar for three analysed forest districts, which is why it was not included as a factor in our experimental design. It additionally suggests that risk effects caused by the wolf presence in the ecosystem may be strong enough to be found even in intensively human-disturbed landscapes.

One might wonder whether the results obtained were affected by differences in the densities of deer within the study areas, especially since the available data indicate a strong relationship between the density of deer and foraging intensity^8,57–59^. However, in the previously mentioned studies, significant differences in foraging intensity were found for deer densities ranging from a dozen to several dozen individuals/km^2^. In contrast, density varied at most in the presented study by only a few individuals/km^2^. Therefore, in our opinion, the effects of deer densities on levels of browsing damage were negligible.

Since the process of forest colonisation by wolves in Poland is relatively fast, it was a challenge to find suitable sites for research. We were able to locate three areas in the same region with similar (although not identical) natural conditions and different statuses of wolf populations in the landscape. Similar studies should include more areas to better analyse the effects of large predators on deer behaviour and forest regeneration, although we believe our research provided satisfactory results. This study suggests how apex predators can alter deer browsing intensity and young tree survival within forest plantations in anthropogenic forest ecosystems through spatial differentiation in the landscape of fear. However, we must remember that the earliest stand stages in commercial forests represent only a part of the total ecosystem and deer browsing pressure may be lower at other stages of forest development. Although the threat posed by human hunters is thought to be the most important determinant of cervid responses in commercial forests during the day through the hunting season^36,60–62^, we have observed a pattern of changes in the distance from the forest edge selection by deer in the face of cursorial predators. We found that open-spaced centres of large-sized clearcuts and young forests, distant from older forest edges, seem to be the safest places for deer to reduce predation risk by wolves and to browse in northern Poland. However, this is not a general rule, as, for example, the results of a study conducted in Scandinavia indicated that such habitats represent the highest risk of predation on moose^61^.

Our study suggests that the presence of wolves significantly affects forest regeneration by influencing the foraging behaviour and browsing of wild herbivores at a fine-scale. The conservation of temperate forests can benefit from the reduction of time deer spend browsing in forest patches with high biodiversity value^63^. Silviculture can benefit from a reduction of time deer spend browsing in forest plantations and wolves can support it. Our results are also relevant for the field of studying cascading effects of predators and how this shapes forests in general. We perceive the wolf as a factor that can reduce and modify the pressure of herbivores at fine-scale and thus help regenerate forests to some extent.

However, the inconsistent results when combining research studies on this topic highlight the need for further research on the cascading effects of large predator populations and human activities on forest regeneration (in both commercial and protected forests). Furthermore, the influence of humans should be considered simultaneously with the influence of natural predators, as it is ubiquitous and cannot be ignored. This could benefit forest management and support the sustainable management of wildlife populations^64^.

## Data Availability

The data that support the findings of this study are available on request from the corresponding author.
